# Quantitative fragmented QRS has a good diagnostic value on myocardial fibrosis in hypertrophic obstructive cardiomyopathy based on clinical-pathological study

**DOI:** 10.1186/s12872-020-01590-2

**Published:** 2020-06-18

**Authors:** Xuanye Bi, Chengzhi Yang, Yunhu Song, Jiansong Yuan, Jingang Cui, Fenghuan Hu, Shubin Qiao

**Affiliations:** State Key Laboratory of Cardiovascular Disease, Fuwai Hospital, National Center for Cardiovascular Diseases, Chinese Academy of Medical Sciences and Peking Union Medical College, Beijing, China

**Keywords:** Quantitative fragmented QRS, Myocadial fibrosis, Hypertrophic obstructive cardiomyopathy

## Abstract

**Background:**

To investigate the relationship between fragmented QRS (fQRS) quantified by a new method and myocardial fibrosis (MF) and the diagnostic value of quantitative fQRS (Q-fQRS) to detect MF in hypertrophic obstructive cardiomyopathy (HOCM) patients based on histological validation.

**Methods:**

We performed a retrospective study that included 69 patients with HOCM who underwent ventricular septal surgery. Nine individuals who died from accidents were studied as a control reference for the histological parameters. Septal myocardium samples were subjected to Masson’s trichrome staining to quantify the collagen volume fraction (CVF). An fQRS pattern was defined as the presence of additional R waves or RSR’, evidenced by notched R or S wave on electrocardiography (ECG). The Q-fQRS was quantified as the total amount of deflections in the QRS complex in all 12 routine ECG leads together. Cardiac magnetic resonance imaging was conducted, and late gadolinium enhancement (LGE) was measured at 2, 4, 6 and 8 standard deviations (SDs).

**Results:**

Of the 69 patients, fQRS was documented in 38 (55.1%) patients, the mean number of leads with fQRS was 3.7 ± 1.6, and the mean Q-fQRS was 17 ± 7.2. Compared with HOCM patients without fQRS, HOCM patients with fQRS had a higher CVF and more LGE at 6 SD (*P* < 0.001; *P* = 0.040). Q-fQRS was correlated with CVF (r = 0.640, *P* < 0.001), and Q-fQRS showed the best correlation with LGE measured at 8 SD (r = 0.379, *P* = 0.002). Multivariate regression analyses revealed that Q-fQRS was independently associated with the extent of CVF in HOCM patients after adjusting for age, sex, body surface area and the extent of LGE at 6 SD (*P* < 0.001). When the patients were divided into subgroups with normal CVF or high CVF according to the CVF in controls, Q-fQRS and LGE at 6SD showed similar diagnostic value in detecting patients with high CVF, with sensitivities of 66.7% vs 68.6%, specificities of 76.7% vs 72.4%, and accuracies of 71% vs 70.3%.

**Conclusions:**

HOCM patients with fQRS showed more extensive MF. Q-fQRS was an independent predictor for MF and had a good diagnostic value, with a sensitivity of 66.7% and specificity of 76.7%, in identifying patients with higher fibrotic burden.

## Background

Hypertrophic cardiomyopathy (HCM) is a common inherited cardiovascular disease that is characterized as myocardial fibrosis (MF) [1]. HCM patients with MF experience left ventricular (LV) adverse remodeling and have a poor prognosis, such as diastolic dysfunction, cardiovascular death, heart failure and fatal arrhythmia [2–6]. MF is a process of collagen deposition that is presently evaluated by endomyocardial biopsy, magnetic resonance imaging (MRI), or circulating biomarkers of collagen turnover. Each detection method has pros and cons.

Late gadolinium enhancement (LGE) is a standard approach for the assessment of MF because collagen deposition and excessive retention of gadolinium lead to enhancement on CMR images [7]. However, due to the limitations and significant costs of advanced techniques, CMR may not always be available. Fragmented QRS (fQRS) is becoming a novel electrocardiogram (ECG) finding and has potential use as a predictor of MF [8]. Qualitative analysis of fQRS has indicated that fQRS can detect myocardial fibrosis, as assessed by the extent of LGE during CMR imaging [9, 10]. A recent study proposed a quantitative measurement of fQRS (Q-fQRS) and showed that Q-fQRS might be an early predictor of arrhythmogenic cardiomyopathy [11]. However, the association between Q-fQRS and MF and the diagnostic value of Q-fQRS remain unknown. Hence, we aimed to investigate the relationship between Q-fQRS and MF based on histologic validation in HOCM patients. Moreover, we also compared the diagnostic value of Q-fQRS to assess the extent of MF between LGE images and Q-fQRS.

## Method

### Study population

Sixty-nine consecutive HOCM patients with symptomatic left ventricular (LV) outflow tract obstruction who underwent surgical myectomy at Fuwai hospital,Beijing between 2015 and 2020 were retrospectively studied. All patients underwent a detailed cardiovascular evaluation, including medical history, clinical examination, 12-lead ECG, and CMR.

HCM was diagnosed by the criteria mentioned previously [12]..LV outflow obstruction was defined as an instantaneous peak doppler LV outflow tract gradient (LVOTG) ≥30 mmHg at rest, or an exercised LVOTG ≥50 mmHg [13]. Patients with obstructive coronary artery disease, history of alcohol septal ablation, history of surgery or trauma within the previous 6 months before the surgery, severe valvular disease,stages 3 to 5 chronic kidney disease (CKD),connective tissue disease and osteoarthropathy were excluded from the study.

Control myocardium from the LV septal wall was collected at autopsy of 9 individuals (6 males,3 females, mean age 45.4 ± 14.3 years) who died from accidents, showing no signs of macroscopic or microscopic cardiac lesions.

### Electrocardiography

A standard 12-lead ECG (0.5–150 Hz, 25 mm/s, 10 mm/mV) was recorded in the supine position during quiet respiration before the surgeries. The fQRS was defined as previously described [14],as follows: in patients with QRS duration < 120 milliseconds, (1) an additional R wave (R prime), (2) notching in nadir of the S wave, (3) notching of R wave, or (4) the presence of more than 1 R prime in 2 contiguous leads corresponding to the left ventricular (LV) segment; in patients with right or left bundle branch block (QRS duration≥120 milliseconds), (1) various RsR’ pattern with > 2 R’, (2) > 2 notches in the R wave, or (3) > 2 notches in the downstroke or upstroke of the S wave, in 2 contiguous leads corresponding to the LV segment. The presence of fQRS in 2 contiguous anterior leads (V1–V5), lateral leads (I, aVL, and V6), or inferior leads (II, III, and aVF) was assigned to detect myocardial fibrosis in the anterior, lateral, or inferior segments, respectively. The Q-fQRS method quantifies the total amount of fragmentation in all 12 routine ECG leads together. The summation of absolute numbers of positive and negative deflection points in each first QRS complex of each ECG lead were labeled as a fQRS count value. The first deviation from the iso-electric line and the last transition from the last deflection to the iso-electric line were excluded [11].

### Echocardiography

Transthoracic echocardiography was performed by an experienced cardio-sonographer using the Phillips iE33 Color Doppler Ultrasound System (Philips Healthcare, Andover, MA, USA). M-mode, twodimensional, and pulsed and continuous-wave Doppler studies were utilized in the standard evaluation according to the guidelines of the American Society of Echocardiography [15]. All patients underwent resting LVOT gradient measurements with continuous-wave Doppler echocardiography, while LVOT gradient after provocation was only determined in those with a resting LVOT gradient < 50 mmHg. The severity of mitral regurgitation was measured with flow Doppler imaging as a qualitative index (mild =1, moderate = 2, moderate to severe = 3, and severe = 4) [16].

### Cardiac MRI

CMR imaging was performed using a 1.5-T speed clinical scanner (Siemens Medical Solutions, Erlangen, Germany) before ventricular septal myectomy. All images were acquired with an electrocardiographically gated breath-hold technique.

To evaluate functional parameters, electrocardiographic gating cine images were then acquired using a segmented, balanced, steady-state-free precession sequence. The images were acquired during multiple short breath holds (8–15 s). After scout images, cine imaging was performed in four chamber, three-chamber and two-chamber long- and short axis views with the following protocol: 6-mm-thick sections with a 2-mm gap between sections, repetition time, 2.7 ms; eho time, 1.2 ms; 70° flip angle; temporal resolution, 40 ms; field of view, 360 × 315 mm2; matrix, 192 × 162 pixels; pixel size, 1.9 × 1.3;slice thickness, 6 mm.

All CMR images were analyzed using standard ventricular analysis software (Argus,VE36A; Siemens Medical Solutions). For all patients, wall thickness at the septal, posterior and LV end-diastolic dimensions were all determined in the short-axis view (at the midpapillary level). Epicardial and endocardial borders of the LV myocardium were manually traced during the whole cardiac phase on each cine short-axis image to obtain LV and RV end-diastolic and end-systolic volumes, ejection fractions, and myocardial mass. Myocardial mass was calculated by multiplying the volume of the myocardium calculated at end-diastole by the specific gravity of the myocardium (1.05 g/ml). The end-diastolic volume index, end-systolic volume index, and mass index were indexed to body surface area. Late Gadolinium Enhanced (LGE) images were obtained 10–15 min after injection of 0.2 mmol/Kg gadolinium-DPTA.The extent of scarred myocardium was determined automatically by computer counting of all hyperenhanced pixels in the myocardium on each of the shortaxis images. Hyperenhanced pixels resembling LGE were defined as those with image intensities of 2SD, 4SD, 6SD, and 8SD above the mean of image intensities in a remote myocardial region in the same image. A percentage of LGE was then generated by the ratio of total LGE mass to total LV mass.

### Cardiac surgery

We applied extended septal myectomy evolving from the classic Morrow procedure. The hypertrophic ventricular septal leading to systolic anterior motion of the anterior mitral valve and LVOT obstruction was resected. The resection range in the long-axis direction started from approximately 4 mm below the aortic ring to the apex of the left ventricle beyond the bases of the papillary muscles. In the shortaxis direction, the myectomy started rightward to the nadir of the right aortic cusp and to the left and terminated near the mitral anterior commissure. Part of the LV anterior free wall detached to the ventricular septal causing LVOT narrowing may also need to be resected. Furthermore, the anomalous chordal attachments between the mitral valve leaflflets or papillary muscle and the ventricular septal were also excised. Additional surgery was performed based on expert consensus among the experienced cardiac surgeons. If intraoperative transoesophageal echocardiography detected a postoperative LVOT gradient > 30 mmHg or more-than-moderate mitral valve regurgitation after weaning from cardiopulmonary bypass, reoperation was required.

### Histologic study

The septal myocardium samples were immediately fixed in 10% buffered formalin and embedded in paraffin. The samples were sectioned and stained with Masson’s trichrome staining for evaluating MF. Four images of every section were acquired with a projection microscope (× 200;Fig. 3). Subsequent image analysis was performed using Image-Pro Plus 6.0 image analysis software (Media Cybernetics Inc., Buckinghamshire, UK). To determine extent of myocardial fibrosis, which was expressed as collagen volume fraction (CVF,%). CVF was calculated as the ratio of collagen-specific staining to the total area of the myocardium in each septal myocardium sample. The endocardium was excluded from analysis. This histological evaluation was performed by two well-trained cardio-pathologists without knowledge of which patient provided the tissue sections.

### Statistical analysis

Continuous variables were expressed as a mean ± standard deviation (SD) or median and interquartile range according to their distribution. Categorical variables were presented as percentage frequency. Differences between groups were compared with the use of the Student t test (for parametric variables) and Mann–Whitney test (for nonparametric variables) for continuous variables. Categorical variables were compared with chi-square tests or Fisher’s exact chi-square tests. The Spearman’s correlation coefficient method was utilized to test for association between continuous variables. Patients were categorized in 2 subgroups according to values of CVF within (subgroup with normal CVF) and above (subgroup with high CVF) the upper limit of normality (established as mean ± 1.96SD obtained in control subjects and equal to 6.09%). Receiver operating characteristic (ROC) analysis was plotted and the accuracy of the ROC curve was determined by measuring the area underneath it (AUC). Sensitivity, specifificity, positive predictive value (PPV), negative predictive value (NPV), and accuracy for the presence of fQRS were defined as previously reported [17]. Multivariate regression analysis was performed using stepwise of the following variables: age, sex, body surface area, Q-fQRS and LGE at 6SD to evaluate if these variables were independently associated with CVF, provided to have a *p* < 0.10 in univariate analysis. This study assessed interobserver reproducibility for CVF by correlation and Bland-Altman analysis. All statistical analyses were conducted by using statistical software package SPSS Statistics V22.0. A value of *P* < 0.05 was considered significant.

## Results

**Fig. 1:**
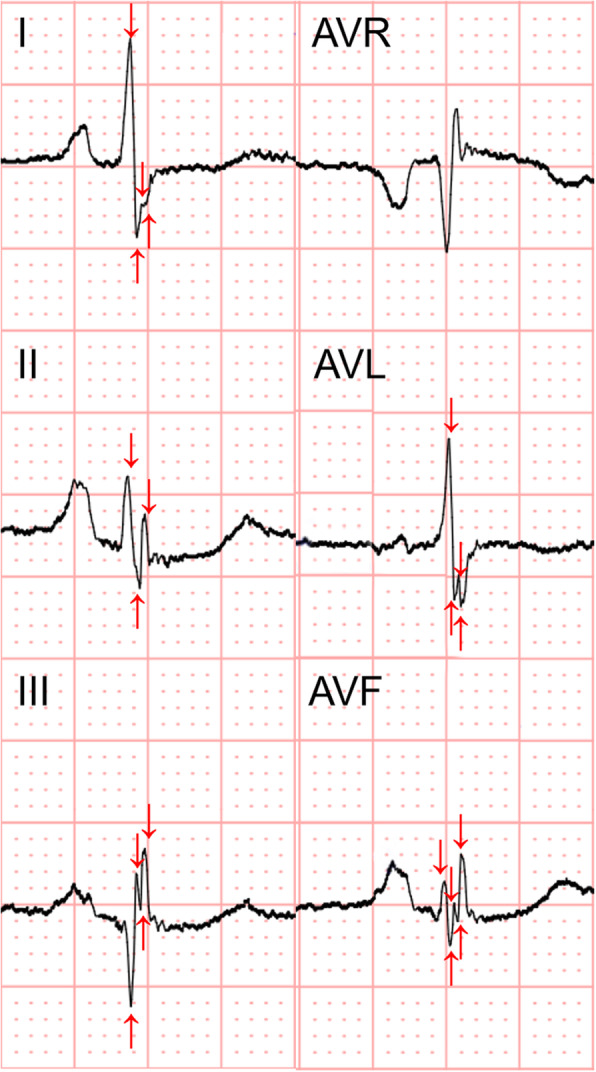
Example of fagmented QRS. The different morphologies of fQRS are shown in this figure; notching of the R wave in leads II, III, and aVF, and notching in nadir of the S wave in leads I, aVL. The quantitative fQRS (Q-fQRS) counting is indicated by red arrows. Each arrow represents a positive or negative deflflection counted as fractionated signal. Lead I, AVL, II, III, AVF have a Q-fQRS count being 4, 4,3, 4 and 5, respectively. The total Q-fQRS count in this figure add up to 20

Sixty-nine HOCM patients who underwent ventricular septal surgery were included, and LGE was assessed in 64 patients. Among the HOCM patients, the mean age was 46.1 ± 14.4 years; 43 (62.3%) were male. fQRS was documented in 38 (55.1%) patients. A representative ECG with fQRS is presented in Fig. 1. The mean number of leads with fQRS was 3.7 ± 1.6. fQRS was observed in the inferior (32/38, 84.2%), anterior (10/38, 26.3%), and lateral (8/38, 21.1%) leads.

The LV mass index, septal thickness, LV end-diastole volume index, LV ejection fraction were all comparable between HOCM patients with and without fQRS (all *P* > 0.05). The extent of LGE at 6SD was higher in patients with fQRS than those without fQRS [6.7%(3.9–10.8%), vs 4.2%(1.6–7.5%), *P* = 0.040]. The baseline characteristics are listed in Table 1.

**Table 1:** Baseline characteristics of 49 patients with HCM

	All patients	Patients with HOCM		*P* value
	(*n* = 69)	With fQRS	Without fQRS	
		(*n* = 38)	(*n* = 31)	
Age,years	46.1 ± 14.4	43.7 ± 15.6	49 ± 12.3	0.129
Male,%	43 (62.3)	22 (57.9)	21 (67.7)	0.401
Dyspnea,%	61 (88.4)	34 (89.5)	27 (87.1)	1
NYHA III/IV,%	22 (31.9)	13 (34.2)	9 (29)	0.646
History of syncope,%	19 (27.5)	13 (34.2)	6 (19.4)	0.169
History of hypertension,%	15 (21.7)	6 (15.8)	9 (29)	0.185
History of diabetes mellitus,%	6 (8.7)	4 (10.5)	2 (6.5)	0.867
Family history of HCM or SCD,%	5 (7.2)	2 (5.3)	3 (9.7)	0.813
NSVT,%	12 (17.4)	7 (18.4)	5 (16.1)	0.803
AF,%	9 (13)	5 (13.2)	4 (12.9)	1
Bundle branch block,%	4 (5.8)	2 (5.3)	2 (6.5)	1
ICD,%	0 (0)	0 (0)	0 (0)	1
SAM,%	68 (98.6)	38 (100)	30 (96.8)	0.449
Mitral regurgitation	2 (1–2)	2 (1–3)	2 (1–2)	0.373
Calcium antagonist,%	25 (36.2)	11 (28.9)	14 (45.2)	0.163
Beta blocker,%	51 (73.9)	33 (86.8)	18 (58.1)	0.007
ACEI/ARB,%	2 (2.9)	0 (0)	2 (6.5)	0.204
Diuretics,%	24 (34.8)	11 (28.9)	13 (41.9)	0.26
CMR				
Septal wall thickness,mm	25 (21–29)	26 (21.8–29)	24 (19–29)	0.637
Left atrium diameter,mm	41.8 ± 7.9	42 ± 8.4	41.5 ± 7.3	0.802
LV end-diastolic diameter,mm	45.6 ± 4.2	46.1 ± 4	44.9 ± 4.3	0.236
LVMI,g/m^2^	88.9 (73.3–117.4)	91.5 (77.2–133.5)	83 (66.9–110)	0.385
LVEF,%	64.8 ± 9.6	64.1 ± 9.6	65.8 ± 9.8	0.47
LVEDVI,ml/m^2^	83.7 ± 20.4	87.7 ± 23.2	78.7 ± 15.4	0.069
LVESVI,ml/m^2^	28.7 (21.8–36.3)	31.7 (23.7–39.4)	25.9 (19.9–30.9)	0.054
LGE at 2SD,%	27.5 (22.4–38.2)	28 (22.5–39.9)	26.9 (19–37.2)	0.403
LGE at 4SD,%	12.8 (6.9–19.1)	14.4 (9.5–21.9)	11.9 (5.8–16.7)	0.067
LGE at 6SD,%	6.2 (1.9–8.7)	6.7 (3.9–10.8)	4.2 (1.6–7.5)	0.04
LGE at 8SD,%	2.4 (0.45–4.65)	3.2 (0.62–5.9)	1.9 (0.33–3.4)	0.057
BSA,m^2^	1.8 (1.6–1.9)	1.7 (1.6–1.9)	1.8 (1.7–1.9)	0.05

Data are presented as the mean value SD (*p* values for 2-sided Student’s t test) or median and interquartile (p values for Mann-Whitney test) or percentage of patients (p values for chi-square test). Volumes are indexed to body surface area. *ACEI* angiotensin-converting enzyme inhibitor, *AF* atrial fibrillation, *ARB* angiotensin receptor blocker, *BSA* body surface area, *ICD* implantable cardioverter-defibrillator, *HCM* hypertrophic cardiomyopathy, *LGE* late gadolinium enhancement, *LVEDVI* Left ventricle end diastolic volume index, *LVEF* left ventricular ejection fraction, *LVESVI* Left ventricle end systolic volume index, *LVMI* left ventricle mass index, *NYHA* New York Heart Association, *NSVT* non-sustained ventricular tachycardia, *SAM* systolic anterior motion, *SCD* sudden cardiac death**,***SD* standard deviation

**Fig. 2:**
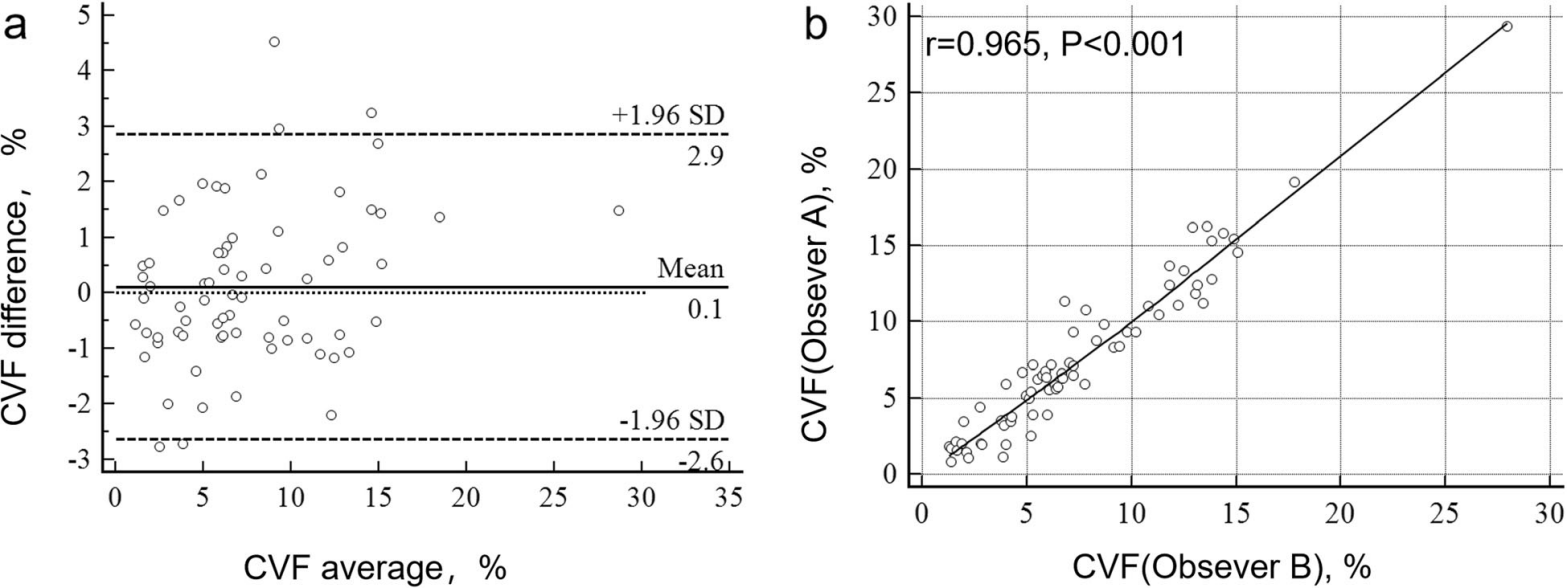
Bland-Altman analysis **a** and correlation **b** of CVF between 2 observers

**Fig. 3:**
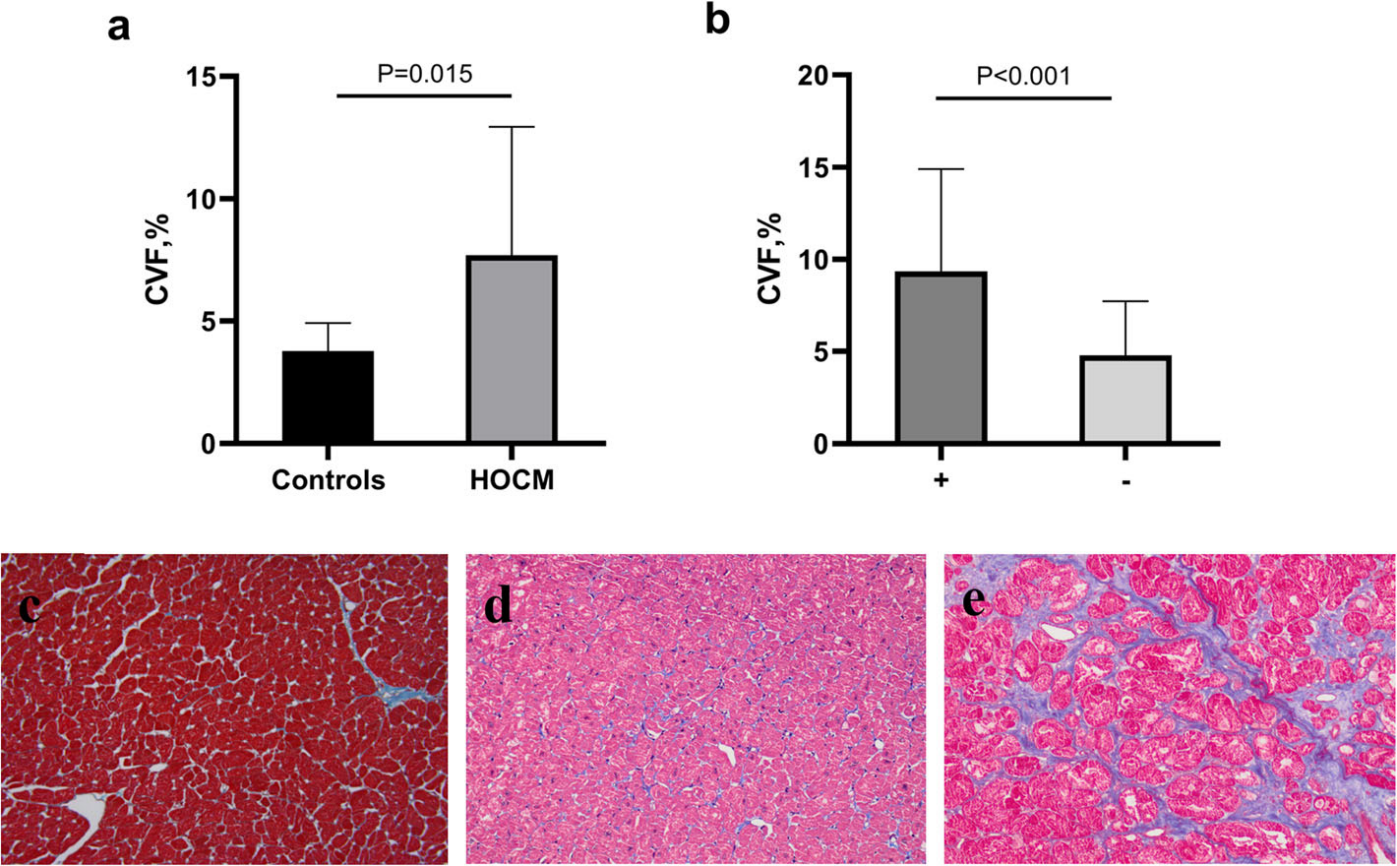
Comparison of CVF in control subjects and HOCM patients and in fQRS(+) and fQRS(−) groups. HOCM patients had higher CVF value than controls **a** (*P* = 0.015). CVF is higher in patients with fQRS than those without fQRS **b** (*P* < 0.001) .Myocardial fibrosis, stained in blue by Masson’s trichrome staining **c**, **d** and **e**. The left panel correspond to mycardial fibrosis in controls **c**, the middle and right panel correspond to mild and severe myocardial fibrosis in HOCM patients **d** and **e**.Magnification×200

Representative images for MF were showed in Fig. 3. Whereas MF were slightly stained in controls (Fig. 3c), it exhibited different levels of expression in HOCM patients (Fig. 3d, e). Bland-Altman analysis showed that the mean difference was 0.1151% and the limit of agreement was − 2.6361-2.8663%(Fig. 2). Both pathologist showed good agreement with CVF values(*r* = 0.965, *P* < 0.001). The mean CVF was higher in patients with HOCM than in controls [6.52% (3.46–11.08%) vs 3.49% (2.90–4.53%), *P* = 0.015, Fig. 3a]. Patients with fQRS showed a higher CVF than patients without fQRS [9.92% (5.89–13.41%) vs 5.16% (1.98–6.47%), *p* < 0.001; Fig. 3b].

**Fig. 4:**
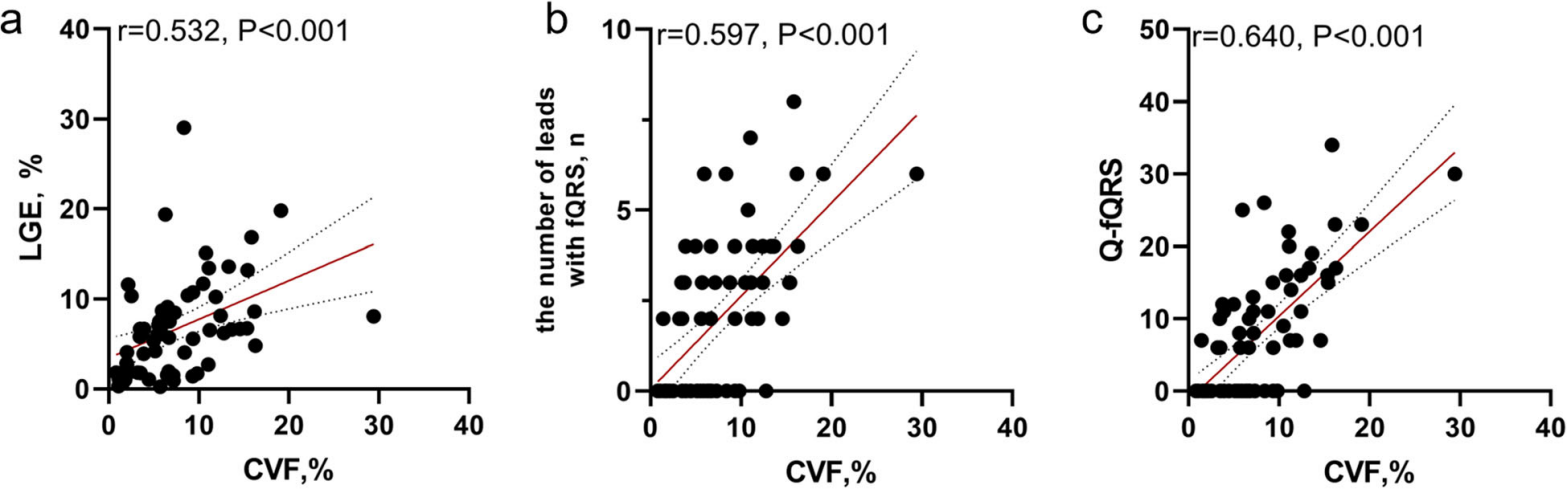
Spearman’s correlation analysis of CVF and clinic parameters. Significance was noted between CVF and LGE at 6SD **a** (*P* < 0.001), the number of lead with fQRS **b** (*P* < 0.001) and Q-fQRS **c** (*P* < 0.001)

In correlation analysis, CVF was correlated with LGE at 2, 4, 6 and 8 SDs (r = 0.265, *P* = 0.033; r = 0.485, *P* < 0.001; *r* = 0.532, *P* < 0.001; *r* = 0.509, *P* < 0.001; Fig. 4a). The number of leads with fQRS showed a positive correlation with CVF (*r* = 0.597 *P* < 0.001; Fig. 4b). The number of leads with fQRS showed the best correlation with LGE measured at 8 SD (*r* = 0.306, *P* = 0.0014). In addition, Q-fQRS was also correlated with CVF (*r* = 0.640, *P* < 0.001; Fig. 4c), and Q-fQRS showed the best correlation with LGE measured at 8 SD (*r* = 0.379, *P* = 0.002). No further correlations were found between fQRS or Q-fQRS and LV structure parameters. Multivariate adjusted regression analyses revealed that Q-fQRS was independently associated with the extent of CVF in HOCM patients after adjusting for age, sex, BSA, and the extent of LGE at 6 SD. See Table 2.

**Table 2:** Predictors of CVF in the HOCM Patients (Unadjusted and Multivariate Regression Analysis)

Variable	Univariate		Multivariate	
	*β ± SE*	*P* value	*β ± SE*	*P* value
Age	−0.043 ± 0.044	0.340	−0.002 ± 0.036	0.953
Sex	−1.470 ± 1.299	0.262	−1.246 ± 1.060	0.244
BSA	−6.015 ± 3.341	0.076		
Q-fQRS	0.412 ± 0.052	< 0.001	0.408 ± 0.067	< 0.001
LGE at 6 SD	0.415 ± 0.113	0.001		

**Fig. 5:**
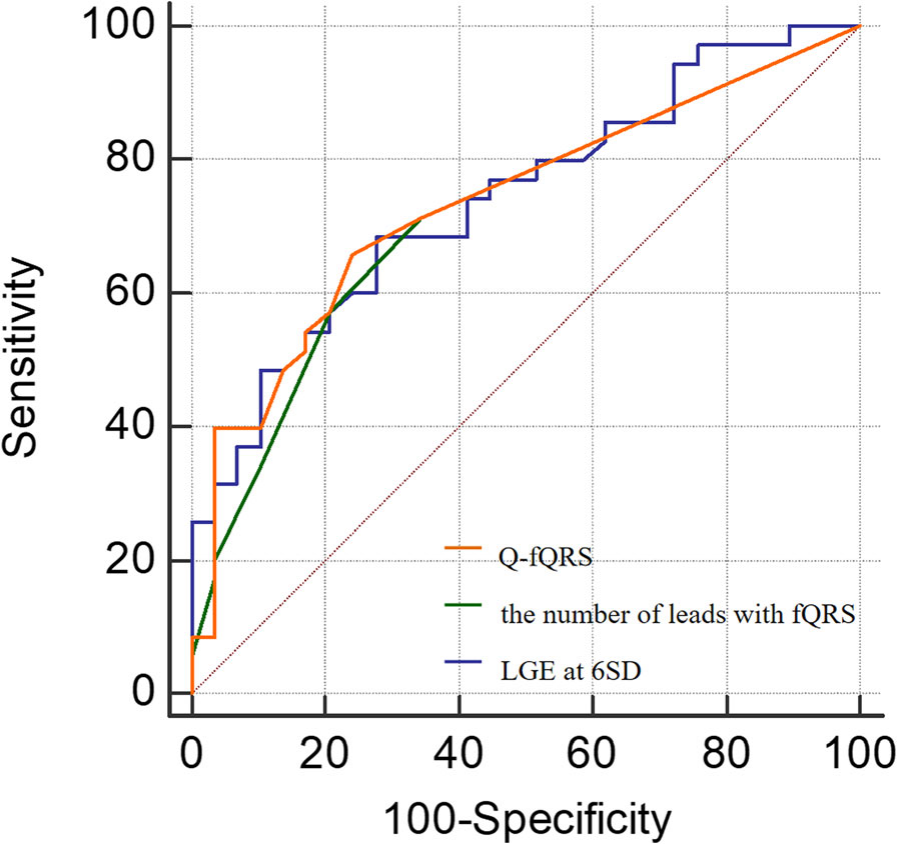
Receiver operating analysis showing the diagnostic value for LGE at 6SD, the number of lead with fQRS and Q-fQRS to identify HOCM patients with high CVF

*BSA* body surface area, *CVF* collagen volume fraction, *LGE* late gadolinium, *Q-fQRS* quantitative fragmented QRS, *SD* standard deviation;

Taking into account the criteria mentioned previously, 30 patients exhibited normal CVF values [3.46% (1.91–5.22%)], and 39 patients exhibited high CVF values [subgroup with high CVF 10.48% (7.16–13.33%)]. In the ROC analysis for the identification of patients with high CVF, the AUC, sensitivity and specificity of Q-fQRS, the number of leads showing fQRS and LGE at 6SD were 0.732 vs 0.715 vs 0.74, 66.7% vs 59% vs 68.6, and 76.7% vs 80% vs 72.4%, respectively. See Table 3, Fig. 5.

**Table 3:** Predictive ability of each examination to discriminate HOCM patients with high CVF from those with normal CVF

	AUC	95%CI	Cutoff value	Sensitivity,%	Specificity,%	PPV,%	NPV,%	Accuracy,%
Q-fQRS	0.747	0.611–0.838	7	66.7	76.7	78.8	63.9	71
The numer of leads with fQRS	0.729	0.594–0.825	3	59	80	79.3	60	68.1
LGE at 6SD	0.741	0.617–0.843	6.22	68.6	72.4	75	65.6	70.3

*AUC* area under curve, *CI* confidence interval, *CVF* collagen volume fraction, *NPV* negative predictive value, *LGE* late gadolinium, *PPV* positive predictive value, *Q-fQRS* quantitative fragmented QRS, *SD* standard deviation;

## Discussion

In this study,we demonstrated the following points: 1. HOCM patients with fQRS exhibited more extensive MF than those with normal QRS wave morphology; 2. Q-fQRS showed good correlation with the fibrotic state of the myocardium and was an independent predictor for MF in HOCM patients; and 3. compared with LGE at 6SD, Q-fQRS showed a similar sensitivity and specificity in identifying patients with severe MF and could be used as an effective and cost-efficient predictor to evaluate fibrotic burden.

MF is one of the main pathological characteristics in HCM patients [17]. MF is associated with diastolic dysfunction, sudden cardiac death and heart failure [4, 18]. Regarding the complex nature of the mechanisms of collagen deposition in HCM, including gene mutation, hemodynamic changes and microvascular rarefaction, the modality of MF varies and develops during the course of disease [19, 20]. Unlike myocardial scars due to acute or chronic myocardial ischemia, MF can either be diffuse or present as multiple patches in HCM [21]. Previous studies showed that MF was mainly comprised of intravascular fibrosis and perivascular fibrosis in HOCM patients [22] and could be induced by old age, volume overload and pressure overload [23]. Although LGE was considered a standard assessment of MF, LGE was less reproducible in HCM than in other conditions. Because of extensive diffuse fibrosis, the areas of LGE were relatively discrete in diffusely abnormal myocardium. Thus, it was difficult to manually delineate the myocardium with a normal signal to set the normal threshold in HCM [24]. Despite good correlations between the extent of LGE and histological fibrosis, there is more to consider, and LGE cannot be simply equated with MF.

Twelve-lead ECG is a simple and cost-efficient tool. Patients with HCM frequently exhibit a wide spectrum of ECG abnormalities, especially in HCM patients with LV outflow track obstruction [25]. fQRS was reported as a novel ECG marker that could be present in many cardiac diseases. The presence of fQRS was correlated with MF and could predict ventricular arrhythmia and poor prognosis [8, 26–28]. A possible explanation for the formation of fQRS is that the increased presence of fibrotic tissue slows activation and results in inhomogeneous depolarization of the ventricles, which probably represents fragmentation in the QRS complex on surface 12-lead ECG. Previous studies have found that fQRS was present in approximately 60% of HCM patients and was correlated with the extent of LGE, which is consistent with our studies [8, 26–28]. More importantly, we demonstrated the correlation between fQRS and the pathological fibrotic state in HOCM patients, supplementing our knowledge about fQRS.

However, fQRS is not limited to patients with cardiac diseases. fQRS is also present in almost 19.7% of subjects without a known cardiac disease and was not associated with increased mortality in these subjects. Although fQRS morphologies could be early markers of subclinical cardiac disease [29], it cannot be ruled out that some fQRS morphologies are benign normal variants, whereas other morphologies represent myocardial scarring. There is a need for a more detailed fQRS classification. Roudijk et al. reported a new approach to quantitate fQRS in patients with arrhythmogenic cardiomyopathy (ACM). With this method, they showed that both definite ACM patients and pathogenic variant carriers without an ACM diagnosis had a higher Q-fQRS than the controls, which might indicate that increased Q-fQRS is an early sign of disease penetrance [11]. In our study, Q-fQRS was correlated with the extent of histological MF in HOCM patients. This indicated that patients with a higher Q-fQRS were more likely to have a higher fibrotic burden. Our study also showed that the diagnostic value of Q-fQRS was good with a sensitivity of 66.7% and specificity of 76.7% in detecting patients with severe MF. Compared with the qualitative definition of fQRS, Q-fQRS exhibited a higher sensitivity and accuracy, indicating that Q-fQRS is an effective and cost-efficient tool to screen for a fibrotic state in HOCM patients.

One limitation of this study was that this was a retrospective study, however, we prospectively enrolled HOCM patients from 2015, and all patients were diagnosed and managed by protocol. Second,because the controls were coming from autopsies of 9 individuals who died from accidents, we had no access to their medical history. Third, sample size was small and restricted to HOCM, which needed to be validated in a larger HCM patient cohort. Fourth, our study limited to single samples of the septum obtained at surgical myectomy, so sampling in different regions might be essential to detect distribution of MF in HOCM patients. Fifth, the lack of long-term longitudinal follow up data prevented us from analyzing the prognostic effect in HOCM patients with high CVF and the influence of collagen accumulation during the development of HOCM.

## Conclusions

In conclusion, HOCM patients with the presence of fQRS showed greater histological MF. Q-fQRS was an independent predictor to detect MF and had a good diagnostic value of 66.7% sensitivity and 76.7% specificity to identify patients with higher fibrotic burden.

## Data Availability

The datasets generated and analysed during the current study are not publicly available due to patient confidentiality but are available from the corresponding author on reasonable request.

## Abbreviations

CVF: Collagen volume fraction
ECG: Electrocardiography
fQRS: Fragmented QRS
HOCM: Hypertrophic obstructive cardiomyopathy
LGE: Late gadolinium enhancement
LV: Left ventricular
MF: Myocardial fibrosis
NPV: Negative predictive value
PPV: Positive predictive value
fQRS: Quantitative fragmented QRS
ROC: Receiver operating characteristic
SD: Standard deviation
